# Epidemiology of Porcine Cysticercosis in Eastern and Southern Africa: Systematic Review and Meta-Analysis

**DOI:** 10.3389/fpubh.2022.836177

**Published:** 2022-03-16

**Authors:** Yewubdar Gulelat, Tadesse Eguale, Nigatu Kebede, Hailelule Aleme, Eric M. Fèvre, Elizabeth A. J. Cook

**Affiliations:** ^1^Aklilu Lemma Institute of Pathobiology, Addis Ababa University, Addis Ababa, Ethiopia; ^2^International Livestock Research Institute, Nairobi, Kenya; ^3^School of Public Health, Addis Ababa University, Addis Ababa, Ethiopia; ^4^Institute of Infection, Veterinary, and Ecological Sciences, University of Liverpool, Liverpool, United Kingdom

**Keywords:** porcine cysticercosis, epidemiology, systematic review, meta-analysis, Eastern and Southern Africa

## Abstract

*Taenia solium* cysticercosis is the most common cause of acquired epilepsy in pig-raising and pork-consuming parts of Africa, Latin America, and Asia. This review aimed to systematically compile and synthesize data on the epidemiology of porcine cysticercosis in the Eastern and Southern Africa (ESA) region. Comprehensive searching strategies were employed to retrieve the studies published or reported between January 1,1997 and March 1, 2021, from Pub Med, Hinari, and Google Scholar databases and search platforms. The identified studies that met the inclusion criteria were then appraised for methodological quality. Finally, 44 studies obtained from nine countries were selected and included in this review. Relevant data were extracted using standardized templates for qualitative synthesis and meta-analysis. The overall pooled prevalence estimate of porcine cysticercosis in the ESA region was 17% (95% CI: 14–20%). The prevalence level between and within countries showed high variability. The pooled estimate showed high heterogeneity among the reports (the inverse variance index value (I^2^) of 98.99%, *p* < 0.05). The meta-analysis sub-grouped by the type of diagnostic test showed the pooled prevalence estimate of 27% (95% CI: 9–50) by carcass dissection; 23% (95% CI: 14–33) by Antibody-based immunodiagnostic techniques; 23% (95% CI: 18-29) by antigen detecting (Ag)-ELISA, 12% (95% CI: 7–18) by meat inspection, and 9% (95% CI: 7-11) by lingual examination. The meta-analysis sub-grouped by region showed a relatively higher pooled prevalence estimate for the Southern region 22% (95% CI: 15–30) compared to 13% (95% CI: 11–15) in the Eastern region. The highest country-based pooled prevalence was obtained from South Africa (33%, 95% CI: 20–48) and Zambia (22%, 95% CI: 16–29), whereas the lowest pooled prevalence was identified in Madagascar (5%, 95% CI: 4-5) and Rwanda (7%, 95% CI: 6–8). The lack of latrine, traditional pig husbandry practices, unprotected water sources, and increase in age were identified as significant risk factors for the occurrence of porcine cysticercosis in the pooled studies. The findings of this review will provide context-specific input to prioritize the possible intervention programs for *T. solium* control in the ESA region. More sensitive and specific test-based prevalence estimates, detailed risk factor investigations, and financial losses analysis are needed to establish feasible control strategies.

**Systematic Review Registration:**

http://www.crd.york.ac.uk/PROSPERO/, identifier: CRD42021238931.

## Introduction

*Taenia solium* cysticercosis is officially recognized as a neglected tropical disease endemic in pig-raising and pork-consuming parts of Africa, Latin America, and Asia (1–4). The lifecycle of *T. solium* involves humans as both the definitive host and an accidental dead-end intermediate host, and pigs as the main intermediate host. Humans acquire the adult *T. solium* through the consumption of undercooked pork infected with cysticerci. Pigs become infected by ingesting tapeworm eggs passed in the stool of the tapeworm carriers during scavenging in the contaminated environment. In humans, accidental ingestion of the tapeworm eggs results in migration and development of the cysticerci in different tissues. The establishment of cysticerci in the brain leads to the development of neurocysticercosis (NCC) (5–8), which is a leading cause of acquired epilepsy in the endemic regions (2). The *T. solium* is ranked among the most important foodborne parasites globally (9). It is responsible for an estimated loss of approximately 2.8 million disability-adjusted life-years majorly due to neurocysticercosis (10).

Pig rearing is an important livelihood activity for many smallholder farmers in sub-Saharan Africa (SSA) (11). The *T. solium* has been reported in almost all countries in the SSA region apart from areas where pig keeping and consumption are not common due to cultural or religious reasons (12–17). Similarly, the traditional pig production and pork consumption have grown fast in the ESA region (15, 18), with the reported case of *T. solium* taeniosis and cysticercosis (TSTC) increasing through time (15, 19). Despite the reported significance of TSTC, it is neglected in most African countries and little effort has been exerted to control or eliminate this neglected zoonotic parasite (15). This led to the establishment of the regional network, the Cysticercosis Working Group for Eastern and Southern Africa (CWGESA), which aimed to improve human health and well-being, as well as the smallholder pig production through facilitating the regional cooperation and sharing of the knowledge and the limited resources (7, 19, 20).

As part of CWGESA, a regional action plan for combating TSCT in the ESA region called the analytical reviews of the existing information at both country and regional level as one focus area to address TSCT (20). Despite the increased reports of TSCT, the compiled overview on its epidemiology at a regional level is still lacking. Hence, this review intends to answer the question, “What are the pooled prevalence, incidence, distribution, and risk factors of *T. solium* cysticercosis in pigs in Eastern and Southern Africa**?**”. The *T. solium* cysticercosis was first indicated as emerging public health and agricultural problem in ESA in the international workshop on taeniasis and cysticercosis held in South Africa in 1997 (19). So, this review aimed to systematically compile and synthesize regional epidemiologic data from 1997 onwards to provide relevant information about the epidemiology of porcine cysticercosis.

## Methodology

### Search Strategy

**Figure 1:**
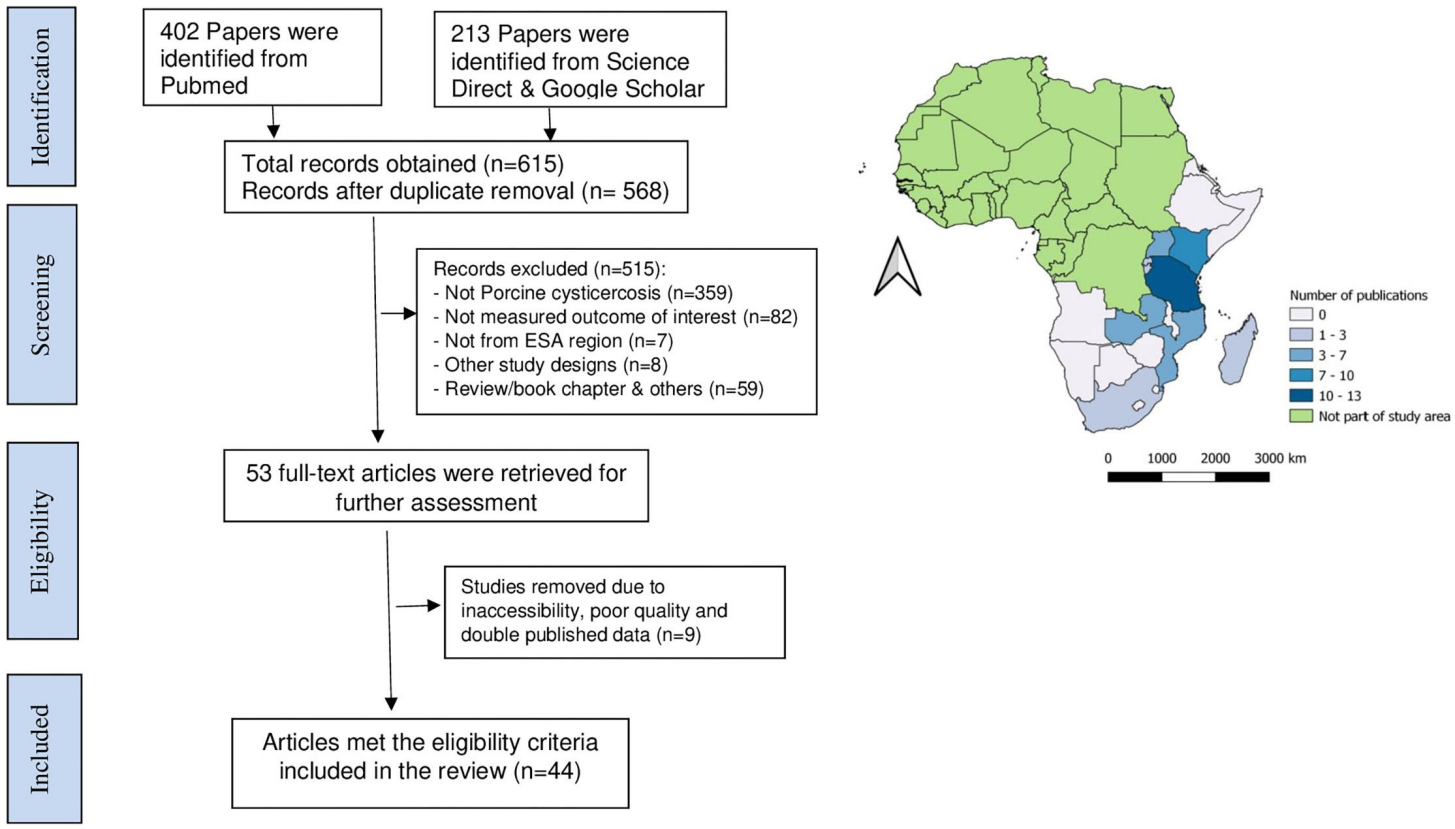
The Preferred Reporting Items for Systematic Reviews and Meta-analyses (PRISMA) flow diagram **(left**) and summary of publications available by country **(right)**.

A systematic review and meta-analysis were conducted following a pre-registered protocol on the International Prospective Register of Systematic Reviews (PROSPERO) database (CRD42021238931) and Preferred Reporting Items for Systematic Reviews and Meta-analyses (PRISMA) guidelines (Supplementary Material 1) to identify relevant articles written in English language and published/reported between January 1, 1997 and March 1, 2021 on the prevalence, incidence, distribution, and risk factors of porcine cysticercosis in ESA. All countries within the ESA region were targeted to search for relevant information about the topic. The ESA was defined as the Eastern and Southern regions of Africa covered by the following countries/territories (Figure 1): Angola, Botswana, Burundi, Comoros, Djibouti, Eritrea, Ethiopia, Kenya, Lesotho, Madagascar, Malawi, Mauritius, Mayotte, Mozambique, Namibia, Réunion, Rwanda, Seychelles, Socotra, Somalia, Somaliland, South Africa, Swaziland, Tanzania, Uganda, Zambia, and Zimbabwe (21).

The search was applied using a three-step search strategy. An initial limited search in PubMed, Health Internetwork Access to Research Initiative (HINARI), and Google scholar was undertaken, followed by an analysis of the text words contained in the titles and abstracts, and of the index terms used to describe the article. A second extensive search was undertaken using identified keywords and index terms across all included databases and search platforms. During the search, the Boolean operators (AND/OR/NOT) were used to combine the mesh terms with the keywords. The mesh terms and keywords used for searching include: “porcine cysticercosis OR *Cysticercus cellulosae* OR *C. cellulosae* OR cysticerc^*^ OR pig tapeworm OR *Taenia solium* cysticercosis OR *T. solium* cysticercosis AND Angola OR Botswana OR Burundi OR Comoros OR Djibouti OR Eritrea OR Ethiopia OR Kenya OR Lesotho OR Madagascar OR Malawi OR Mauritius OR Mayotte OR Mozambique OR Namibia OR Réunion OR Rwanda OR Seychelles OR Socotra OR Somalia OR Puntland OR Somaliland OR South Africa OR Swaziland OR Tanzania OR Uganda OR Zambia OR Zimbabwe”. Then, the reference lists of studies included in the reviews were hand-searched for further eligible studies. The references from the search in each database were imported directly into EndNote citation manager X6.

### Selection Criteria

The predefined inclusion and exclusion criteria were used to screen the relevance of titles and abstracts for this review. The studies about *T. solium* cysticercosis were included in the review if they recruited pig as a study animal, employed a cross-sectional or cohort study designs, conducted within Eastern and Southern Africa region, reported porcine cysticercosis prevalence (number of infected pigs/ total number of pigs examined/tested) and/or incidence (number of infected pigs /pig-time), mentioned the diagnostic methods used, written in English, and published within a period between January 1,1997 and March 3,2021.

### Study Selection

Following the search, all identified citations were collated and uploaded into EndNote citation manager X6 and duplicates were removed. The titles and abstracts were then screened against the inclusion criteria. Those studies meeting the eligibility criteria were retrieved in full. The full texts of selected studies were assessed in detail, and those that did not meet the inclusion criteria were excluded. Included studies underwent a process of critical appraisal. Any disagreements were resolved through discussion with the two primary reviewers and a third reviewer. The result of the search and summary of publications available by country is presented below (Figure 1).

### Data Extraction

Relevant data were extracted from the papers that included the review using a standardized data extraction template developed using a Microsoft Excel workbook. Double data extraction and entry were performed to ensure accuracy. The variables extracted from each article were: name of the journal, title of the article, first author, publication date, country, study location, study period, study design, sample size, diagnostic methods, number of subjects with positive test results, the degree of association between the outcome of interest with each predictor variable (Odds ratio and 95% confidence interval was extracted for each risk factor). The authors of the papers were contacted to request missing or additional data if required. Any disagreements were resolved through discussion and a third reviewer.

### Assessment of Methodological Quality

The selected studies were critically appraised by two independent reviewers using the standardized critical appraisal instruments: (1) the Newcastle Ottawa Quality Assessment Scale for cohort studies, and (2) the Newcastle Ottawa Quality Assessment Scale adapted for cross-sectional studies (22) were employed to guide the quality assessment of the included studies. Any disagreements were resolved through discussion and a third reviewer. Details on the critical appraisal assessment result for the selected studies is provided in Supplementary Material 5.

### Data Analysis

The articles were, as much as possible, pooled in a statistical meta-analysis. and the pooled prevalence (%) of porcine cysticercosis and odds ratio of significant risk factors with their 95% confidence intervals (CI) were calculated. Ninety-five percent of exact binomial CI was calculated for every prevalence. The studies were stratified based on the type of study design, diagnostic methods, country, and region, and a separate meta-analysis was conducted when sufficiently reported data were available (>2 studies). Forest plots were presented for proportions of individual studies, sub-group, and overall prevalence. Heterogeneity among the included studies was assessed using the I-squared test and Q statistic *(P-*value 0.1). The random-effect model was used for the meta-analysis. Sensitivity analyses were performed by assessing the influence of omitting a single study on the overall estimate. A funnel plot and the Egger's regression assumption were used to investigate publication bias. If statistical pooling is not possible, the findings are presented in the narrative form including tables and figures to appropriately aid in data presentation. The analysis of the data was conducted using the STATA statistical software package.

## Result

A total of 615 articles were obtained from all data sources. After the removal of duplicates (23), 568 article titles and abstracts were screened, and 515 records were excluded following predefined selection criteria (Figure 1). Then, 53 records were passed for full article reading, of which nine were excluded because two contained duplicate data, six were inaccessible, and the other did not pass the quality assessment. Finally, 44 full-text articles met the predefined inclusion criteria and passed the quality assessment for meta-analysis, and were included in the qualitative synthesis.

**Figure 2:**
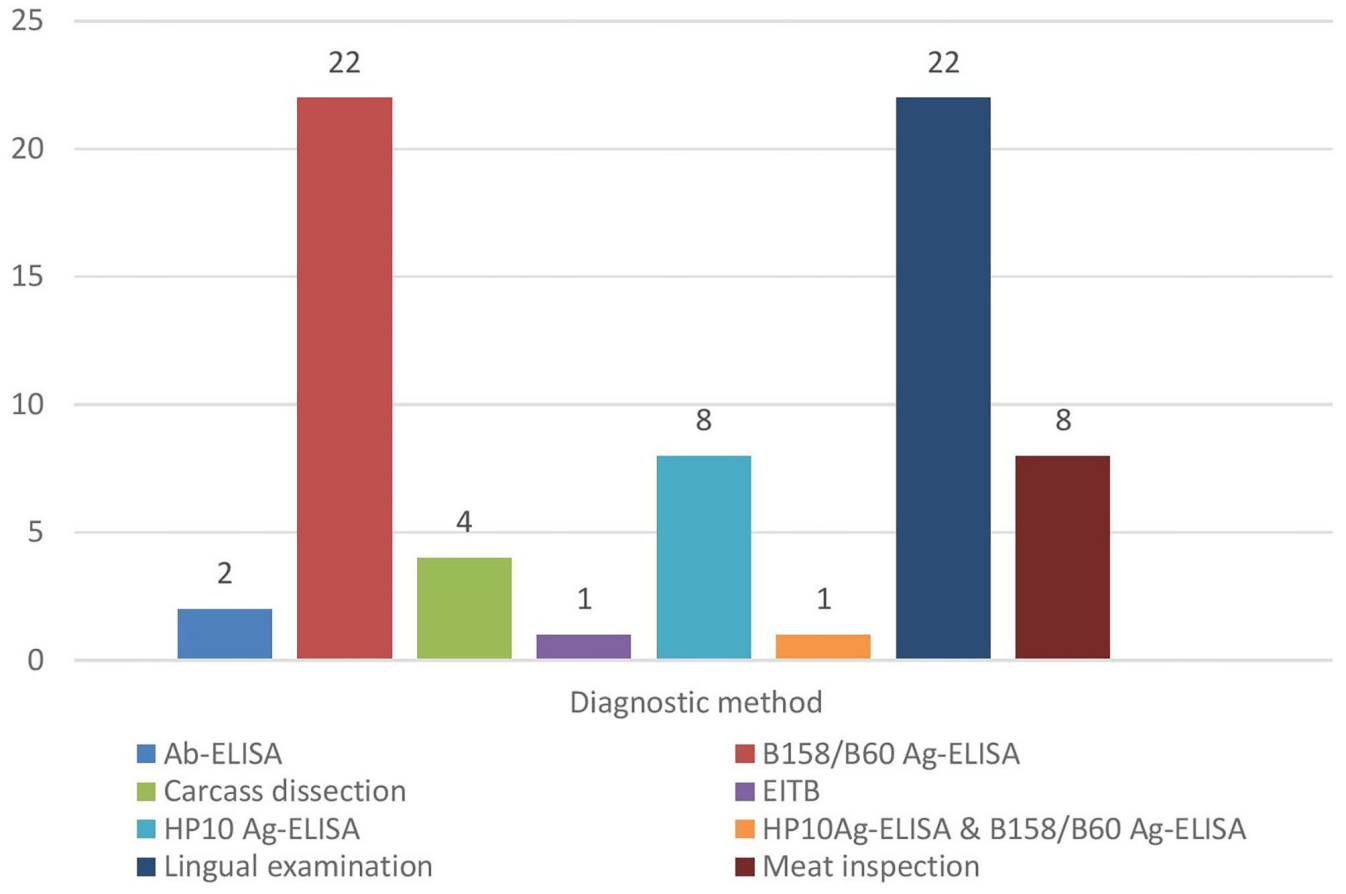
Type of diagnostic techniques employed in the reviewed studies.

Out of the 27 countries/territories studied, the records that met the eligibility criteria were obtained from nine countries (Figure 1). Of these, most records were obtained from Tanzania (*n* = 13) and the others included data from Kenya (*n* = 9), Uganda (*n* = 4), Rwanda (*n* = 1), Burundi (*n* = 2), South Africa (*n* = 3), Zambia (n = 6), Mozambique (*n* = 5), and Madagascar (*n* = 2). From the 44 studies included in the review, 15 employed more than one diagnostic technique. Most studies included in the review used Ag-ELISA (B158/B60 Ag-ELISA and HP10 Ag-ELISA) (24) to ascertain cases of porcine cysticercosis, followed by lingual examination (22), meat inspection (8), carcass dissection (4), and antibody-based immunodiagnostic techniques [Enzyme-linked Immunoelectro Transfer Blot (EITB) and Ab-ELISA] (3) (Figure 2).

The available prevalence data identified through a review of cross-sectional studies are summarized in Tables 1, 2. For each included study, authors, year of publication, the number of sampled pigs, and the prevalence of porcine cysticercosis based on immunological and parasitological diagnostic techniques along with 95% CI are reported.

**Table 1:** Prevalence of porcine cysticercosis based on immunological diagnostic techniques.

**Reference**	**YOP**	**Country**	**Sample size**	**Prevalence of PCC and 95% CI**	
				**Ag–ELISA**	**Ab-assay**
Akoko et al. (25)	2019	Kenya	700	8.7^a^ (6.7–11.1)	
Braae et al. (26)	2014	Tanzania	822	15.5^a^(13.1–8.1)	
Chembensofu et al. (27)	2017	Zambia	68	52.9^a^(40.4–65.2)	
Chilundo et al. (28)	2017	Mozambique	262	12.6^a^(8.8–17.2)	
Dorny et al. (29)	2004	Zambia	868	57.1^a^(53.8–60.5)	24.9^c^(22-27.9)
Eshitera et al. (30)	2012	Kenya	232	32.8^b^(26.8–39.2)	
Fèvre et al. (31)	2017	Kenya	91	17.6^b^(10.4–27)	
Kabululu et al. (24)	2020	Tanzania	350	19.4^a^(15.4–24)	
Kabululu et al. (32)	2015	Tanzania	482	11.4^a^(8.7–14.6)	
Kagira et al. (33)	2010	Kenya	284	3.9^a^(1.9–6.8)	
Komba et al. (34)	2013	Tanzania	600	18.9^a^(27.6–35.2)	
Krecek et al. (35)	2008	South Africa	261	54.8^a^(48.5–60.9) 40.6^b^(34.6–46.8)	33.3^d^(27.6–39.4)
Krecek et al. (36)	2012	South Africa	256	41^a^ (34.9–47.3) 54^b^ (47.6–60.1)	
Kungu et al. (37)	2017	Uganda	1,185	12.2^e^(10.3–14.1)	
Thomas (38)	2013	Kenya	93	17.2^b^(10.2–26.4)	
Maganira et al. (39)	2019	Tanzania	447	17.2^a^(13.8–21.1)	
Matos et al. (40)	2011	Mozambique	132		12.1^c^(7.1–18.9)
Nguhiu et al. (41)	2017	Kenya	276	4.3^a^(2.3–7.5)	
Nsadha et al. (42)	2014	Uganda	378	25.7^a^(21.3–30.4)	
Phiri et al. (43)	2002	Zambia	249	13.7^a^(9.6–18.6)	
Pondja et al. (14)	2010	Mozambique	661	34.9^a^(31.3–38.7)	
Pondja et al. (44)	2015	Mozambique	108	5.6^a^(2.1–11.7)	
Shongwe et al. (45)	2020	South Africa	126	7^a^ (3.3–13.1)	
Porphyre et al. (46)	2015	Madagascar	175	10.9^a^(6.7–16.4)	
Shonyelaet al. (47)	2017	Tanzania	330	33.3^a^(28.3–38.7)	
Sikasunge et al. (48)	2007	Zambia	800	37.6^a^(34.3–41.1)	
Sikasunge et al. (49)	2008	Zambia	1,691	23.3^a^(21.3–25.4)	
Thomas et al. (17)	2016	Kenya	343	49.9^b^(44.4–55.3)	
Waiswa et al. (13)	2009	Uganda	480	8.5^a^(6.2–11.4)	
Wardrop et al. (50)	2015	Kenya	93	17.2^b^(10.2–26.4)	

*YOP, Year of publication; PCC, porcine cysticercosis; CI, Confidence Interval; Ag-ELISA, Antigen based ELISA; Ab-assay, Antibody-based immunodiagnostic techniques; a, Results obtained from B158/B60 Ag-ELISA; b, Results obtained from HP10 Ag-ELISA; c, Results obtained from Ab-ELISA; d, Results obtained from EITB; e, Results obtained from HP10Ag-ELISA & B158/B60 Ag-ELISA*.

**Table 2:** Prevalence of porcine cysticercosis based on Lingual examination, meat inspection, and carcass dissection methods.

**References**	**YOP**	**Country**	**Sample size**	**Prevalence of PCC and 95% CI**		
				**LE**	**MI**	**CD**
Boa et al. (51)	2006	Tanzania	1,832	9.5 (8.2–10.9)		
Chembensofu et al. (27)	2017	Zambia	68	5.9 (4.1–6.5)		55.9 (43.3–67.9)
Dorny et al. (29)	2004	Zambia	868	13.2 (11.1–15.7)	13.9 (11.7–16.4)	
Eshitera et al. (30)	2012	Kenya	392	5.6 (3.6–8.4)		
Kabululu et al. (24)	2020	Tanzania	350			8.3 (5.6–11.7)
Kabululu et al. (52)	2020	Tanzania	282			9.2 (6.1–13.2)
Komba et al. (34)	2013	Tanzania	600	8.8 (6.7–11.7)		
Krecek et al. (35)	2008	South Africa	261	11.9 (8.2–16.4)		
Thomas (38)	2013	Kenya	93	9.2 (4.5–17.6)		
Minani et al. (53)	2021	Burundi	496	15.5 (12.4–19)		
Mkupasi et al. (54)	2011	Tanzania	731		5.9 (4.3–7.8)	
Mushonga et al. (55)	2018	Rwanda	984^LE^/1,720^MI^	3.86 (2.7–5.2)	9.2 (7.9–10.7)	
Mutua et al. (56)	2007	Kenya	505	6.5 (4.5–9.1)		
Newell et al. (57)	1997	Burundi	81		16 (8.8–25.9)	
Ngowi et al. (23)	2010	Tanzania	784	7.3 (5.5–9.3)		
Ngowi et al. (58)	2004	Tanzania	770	17.4 (14.8–20.3)		
Ngowi et al. (59)	2004	Tanzania	70	0 (0–0.5)		
Phiri et al. (43)	2002	Zambia	1,316^FB^	10.9 (9.2–12.7)	20.6 (18.4–22.9)	
Phiri et al. (43)	2002	Zambia	249	6.4 (3.7–10.2)		
Phiri et al. (60)	2006	Zambia	65	7.7 (2.5–17)	18.5 (9.9–30)	47.7(35.1–60.5)
Pondja et al. (14)	2010	Mozambique	661	12.7 (10.3–15.5)		
Porphyre et al. (61)	2015	Madagascar	68,432^FB^		4.7 (4.5–4.8)	
Shonyelaet al. (47)	2017	Tanzania	698	6.3 (4.6–8.4)		
Sikasunge et al. (48)	2007	Zambia	800	18.8 (16.1–21.6)		
Sikasunge et al. (49)	2008	Zambia	1,691	10.8 (9.4–12.4)		
Thomas et al. (17)	2016	Kenya	343	5.5 (3.4–8.5)		
Yohana et al. (62)	2013	Tanzania	308	7.5 (4.8–11)		
Zirintunda and Ekou (63)	2015	Uganda	178		18 (12.6–24.4)	

*YOP, Year of publication; PCC, porcine cysticercosis; CI, Confidence Interval; LE, Lingual examination; MI, Meat inspection; CD, Carcass dissection; FB, facility-based study*.

**Figure 3:**
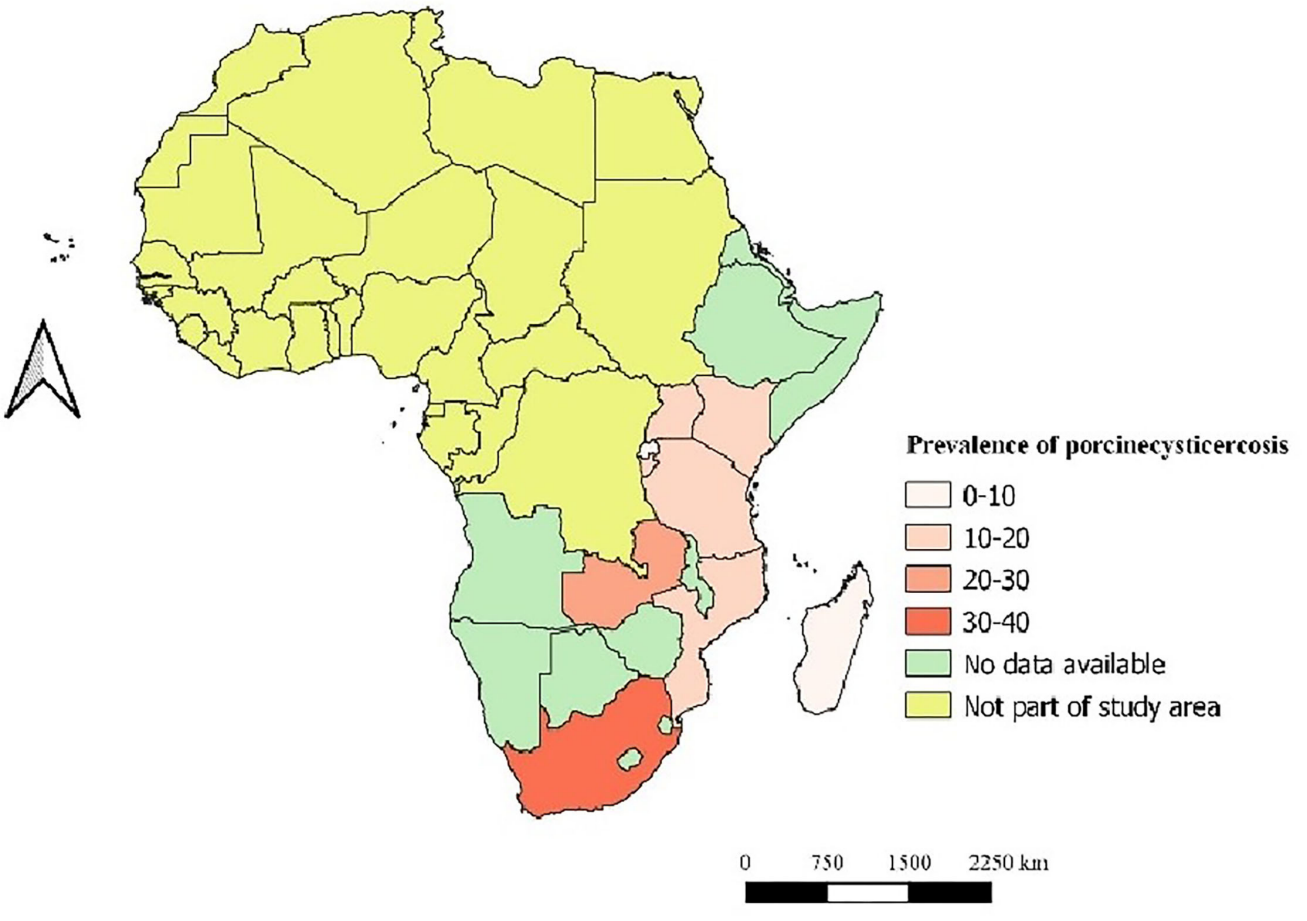
The distribution of porcine cysticercosis in the Eastern and Southern Africa (ESA) region based on the reviewed studies.

This review identified high variability in the prevalence levels among and within countries ranging from 0 to 57% using different diagnostic techniques (Figure 3). Since few studies employed the gold standard techniques to ascertain cases of porcine cysticercosis, we included the studies if they clearly described the employed diagnostic technique. For all the studies, we computed prevalence by dividing the number of positive cases by the total number of pigs tested. Though we did not consider the sensitivity and specificity of used techniques in the prevalence estimation, we assessed the effect of diagnostic test parameter variation by undertaking a sub-group meta-analysis. Pondja et al. (44) in Mozambique reported the point prevalence (only the estimate from the first prevalence survey was included in Table 1) and the incidence rate (the mean incidence rate of 6.2 cases per 100 pig-months between 4 and 9 months of age, and 21.2 cases per 100 pig-months between 9 and 12 months of age) using B158/B60 Ag-ELISA. As this report was the only cohort study obtained, so far, it was only included in the qualitative synthesis.

**Figure 4:**
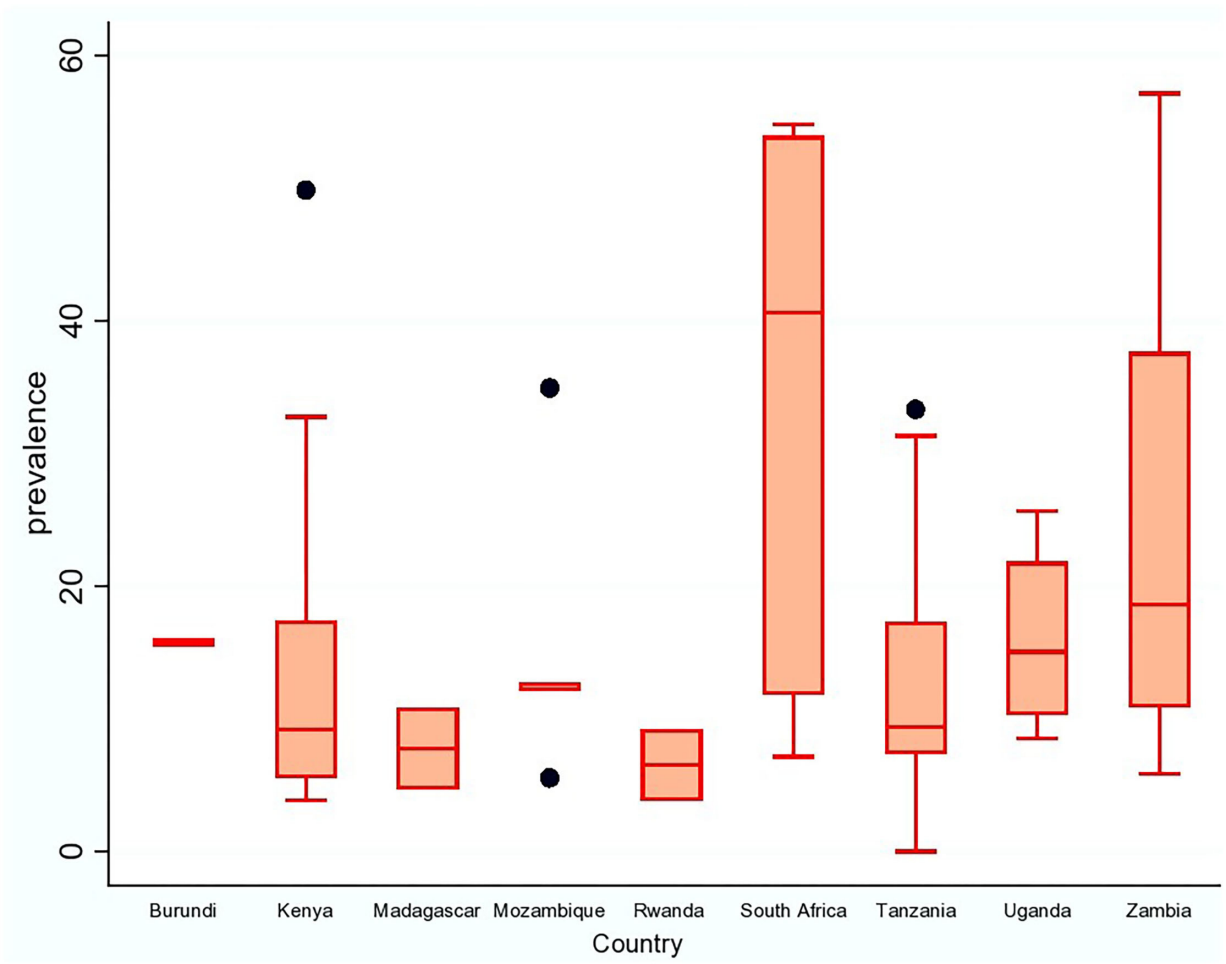
Prevalence of porcine cysticercosis in countries within ESA region.

Figures 3, 4 demonstrate the high variation in prevalence range between countries as well as within countries. The distribution of porcine cysticercosis in the ESA region is provided in Figure 3, where each color represents the average prevalence range classified into 0–10%; 10–20%, 20–30%, and 30–40%. From 27 countries/territories of the ESA region, no article was obtained from 18 countries/territories. At the regional level, South Africa is the country with the highest average prevalence range (30–40%), whereas Rwanda and Madagascar reported lower average prevalence (0–10%). Zambia reported the highest point prevalence (57%), whereas the lowest point prevalence (0%) was reported from Tanzania. Similarly, high prevalence variation was observed within-country reports which are evident with a wide 95% confidence interval in South Africa and Zambia, and the presence of outlier values in Tanzania, Mozambique, and Kenya (Figure 4).

**Figure 5:**
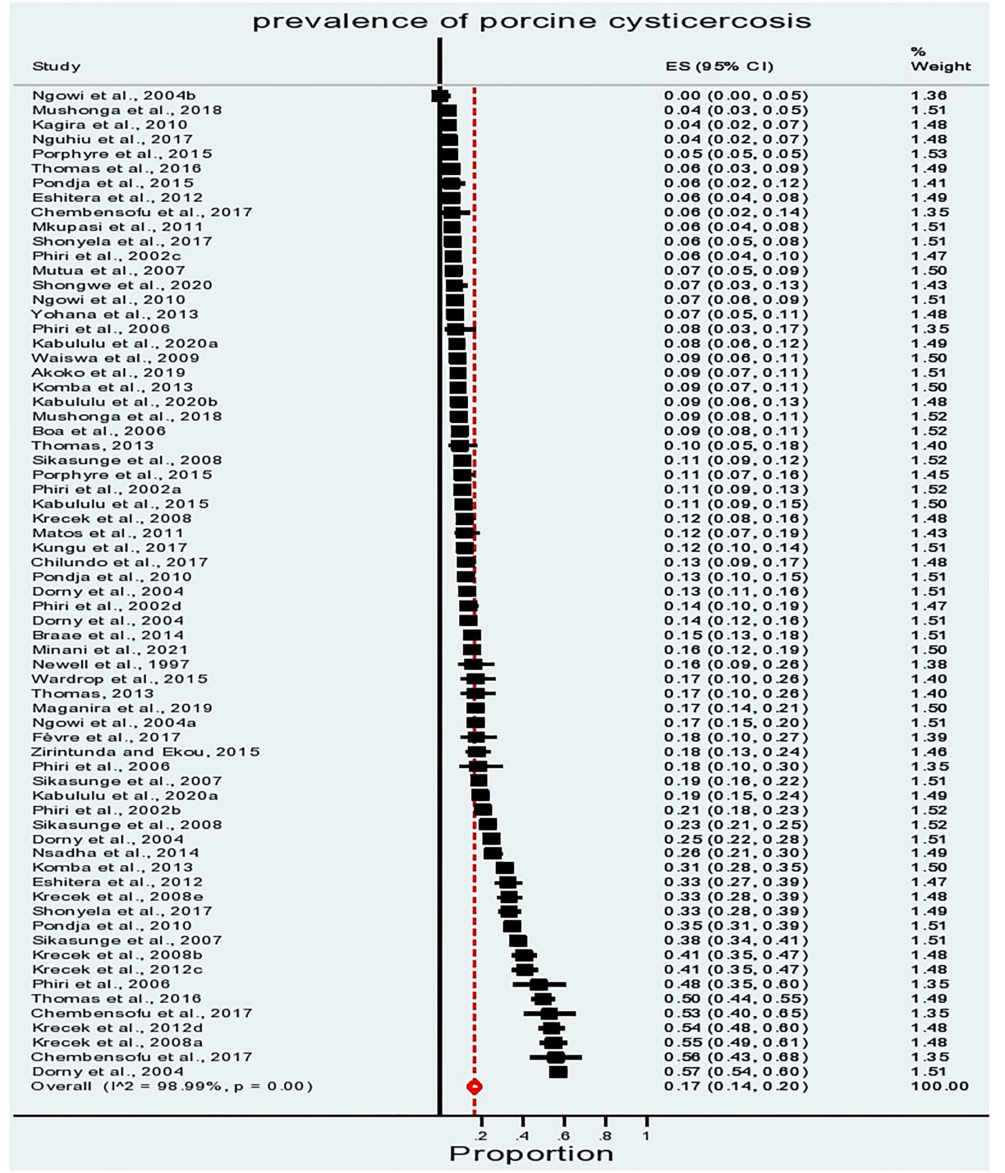
Forest plot showing the studies reporting porcine cysticercosis in the ESA region. The box shows the weight and estimate of the study; the length of the horizontal lines indicates the 95% CI; the vertical broken red line indicates the pooled estimate; the diamond-shaped box at the bottom represents the 95% CI; the solid line indicates the point of null assumption.

The overall pooled prevalence estimate of porcine cysticercosis in the ESA region was 17% (95%CI: 14–20%) (Figure 5). The sensitivity analysis was performed to examine the influence of a single study on the overall estimate and omitting a single study in the analysis did not show a significant impact on the overall estimates (Supplementary Material 2). The calculated Cochran Chi-square value (Q statistic) (*P*< *0*.001) and the inverse variance index value (I^2^) of 98.99% indicate a high degree of heterogeneity among the reports (Figure 5). Moreover, the funnel plot analysis showed the significant effect of small studies (*p*< *0*.005) and the presence of publication bias (Supplementary Material 3).

**Figure 6:**
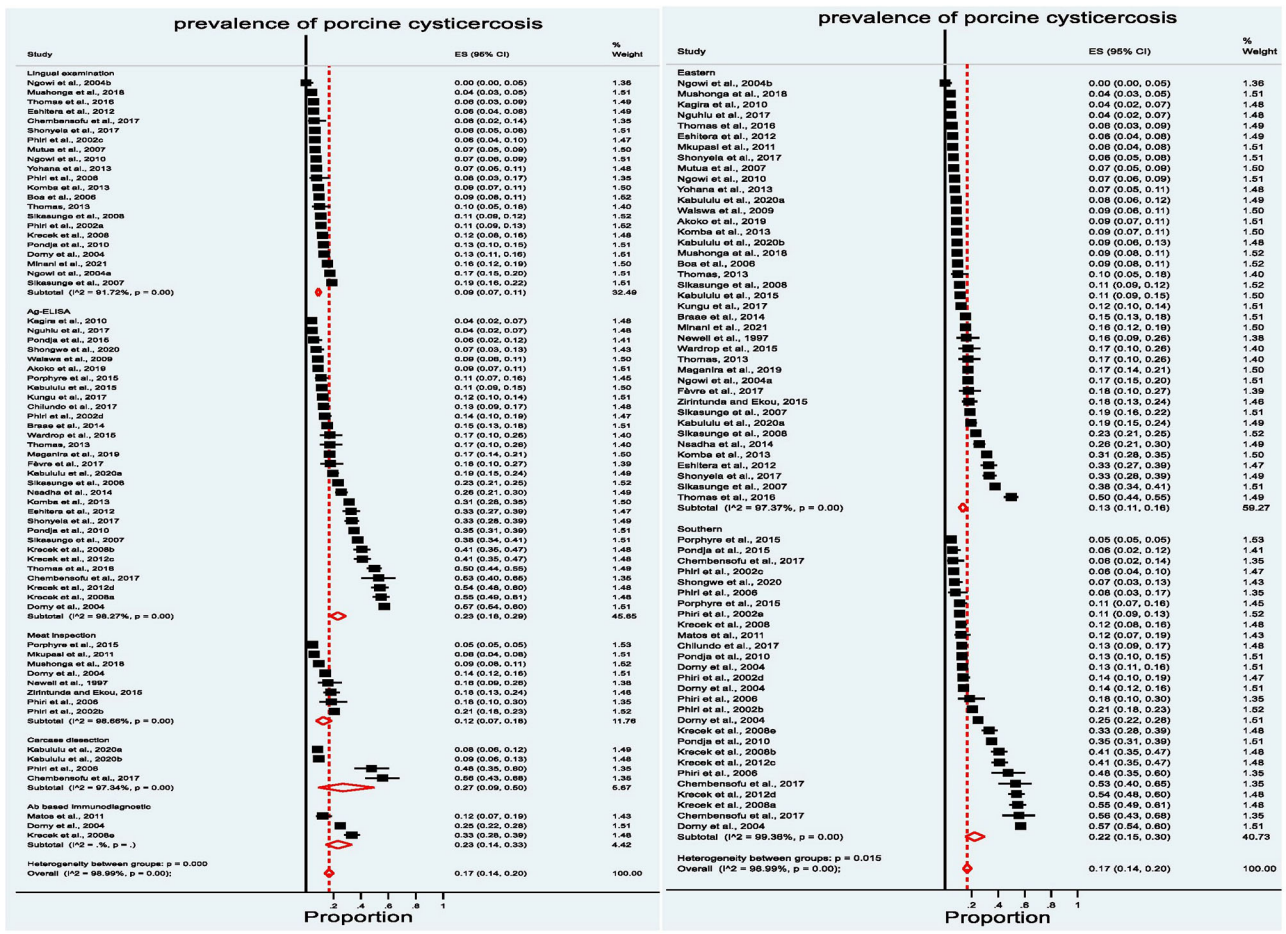
Forest plot showing subgroup analysis of prevalence reports grouped by the diagnostic technique **(left)** and region **(right)**.

Since the overall prevalence estimate showed high variation among the reports, a subgroup analysis was performed based on the country, the types of diagnostic technique, and the region (Eastern and Southern). The results of the sub-group analysis are presented in a forest plot in Figure 6 and Supplementary Material 4.

The meta-analysis sub-grouped by the diagnostic test showed the pooled prevalence estimate of 27% (95% CI: 9–50) for studies employing carcass dissection, 23% (95% CI:14–33) for studies that used Antibody-based immunodiagnostic techniques, 23% (95% CI: 18–29) for those employing Ag-ELISA, 12% (95% CI: 7–18) using meat inspection, and 9% (95% CI: 7–11) by lingual examination. The meta-analysis sub-grouped by region showed a relative difference in the pooled prevalence estimate, which is higher for the Southern region with 22% (95% CI: 15–30) compared to 13% (95% CI: 11–15) for the Eastern region. The sub-grouped prevalence based on country estimated the highest pooled prevalence from South Africa (33%, 95% CI: 20–48) and Zambia (22%, 95% CI: 16–29). Burundi, Uganda, and Mozambique reported a 15% pooled prevalence (within 95% CI range of 6–26%), followed by Kenya at 13% (95% CI: 7–21) and Tanzania at 12% (95% CI: 9–16). Madagascar (5%, 95% CI: 4–5) and Rwanda (7%, 95% CI: 6–8) reported the lowest pooled prevalence (Supplementary Material 4). The high I^2^ values (> 97%) for each of the subgroup analyses indicate a high degree of heterogeneity between studies applying a similar methodology, within and among countries, and sub-regions.

**Figure 7:**
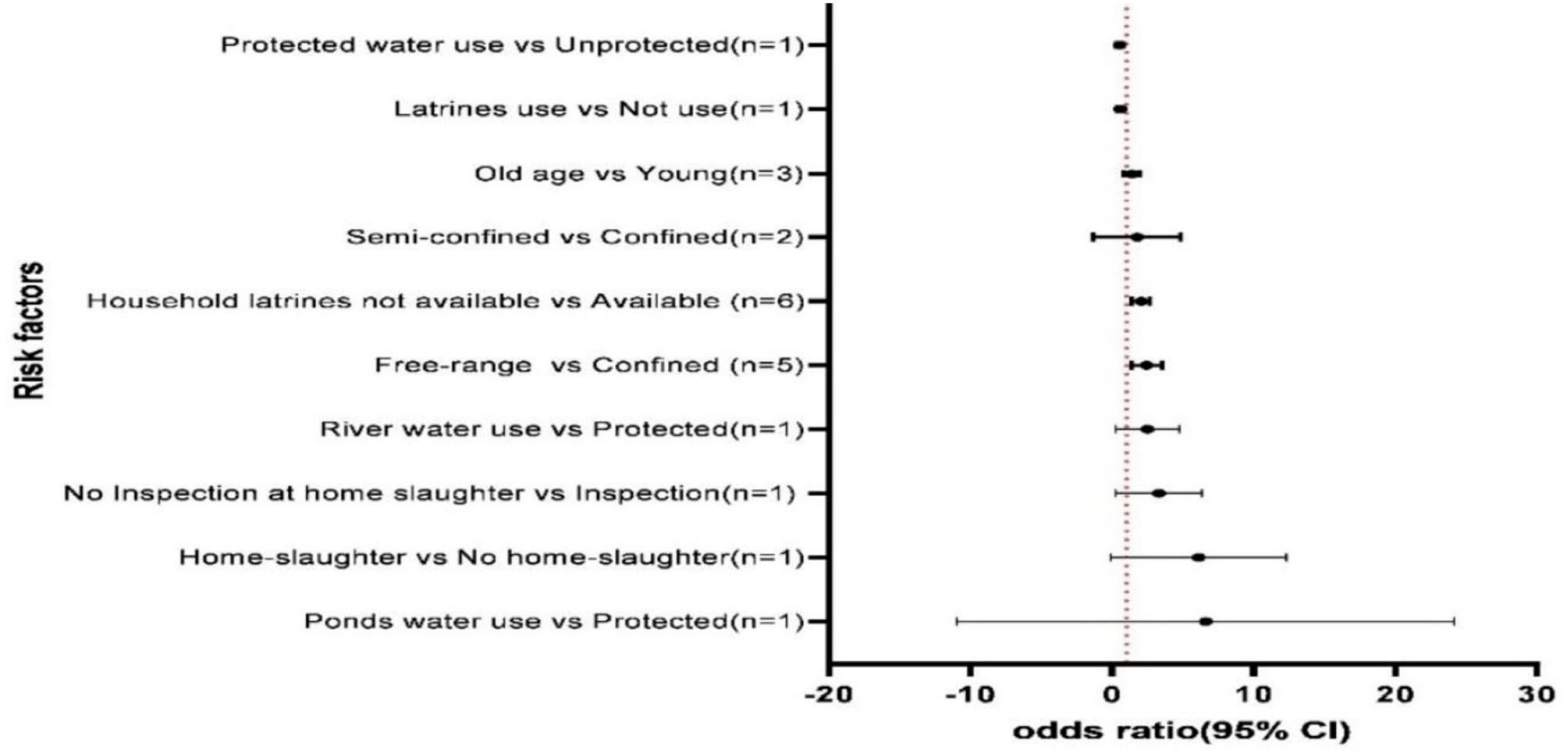
Overview of the meta-analyses results of pooled odds ratio (OR) for significant risk factors of porcine cysticercosis in ESA (n, number of studies that are included in the analysis).

Thirteen articles out of the 44 selected studies reported statistically significant risk factors for the occurrence of porcine cysticercosis. To be significantly associated with porcine cysticercosis in ESA, the identified risk factors were as follows: lack of latrine at household level *(n* = 6); keeping free-range pigs (*n* = 5); semi-confined pig management (*n* = 2); home-slaughter (*n* = 2); unprotected water source (water obtained from rivers, streams, wells, lakes, ponds, and so on) (*n* = 3), and older age of pigs (*n* = 3). The pooled OR of 2.4 was recorded for keeping free-range pigs with low heterogeneity (I^2^ = 55.7%, *p* = 0.06), an OR of 2 was recorded for lack of latrine at household level with no heterogeneity (I^2^ =0%, *p* = 0.72), showing homogeneity among reports. The I^2^ values for old age and semi-confined pig management system showed higher heterogeneity I^2^>72.9 (*p*< *0*.05). The summary of the pooled OR between the studied variables and porcine cysticercosis is shown in Figure 7.

## Discussion

The present study aimed to review 24 years of published literature on the epidemiology of porcine cysticercosis in the ESA region. Out of 27 countries/territories studied, records that met the eligibility criteria were obtained from only nine countries. However, the absence of data for some countries does not exclude the possibility that this parasite is present there. The distribution of TSTC in SSA was reported as not well-documented and under-reported (15, 38).

The overall pooled prevalence of porcine cysticercosis based on the included studies was 17% (95% CI: 14–20%). Despite the poor diagnostic accuracy of tests used in most of the included studies, the presumptive investigation of the primary studies suggests the presence of *T. solium* in the region. Africa is one of the regions conducive for maintaining the full cycle of *T. solium* because of the favorable conditions and other factors associated with poverty (23). The seroprevalence of *T.solium* taeniasis and human cysticercosis in Africa have been estimated to be 0–17.25% and 0.68–34.5%, respectively (16).

This review identified high variability in the prevalence of porcine cysticercosis between and within countries ranging from 0 to 57%. In country-based sub-group analysis of the ESA region, the highest pooled prevalence (33%, 95% CI: 20–48) was obtained from South Africa. Based on the data obtained from Food and Agriculture Organization Corporate Statistical Database (FAOSTAT), the country is among the top countries in ESA in terms of the number of pigs between 1997 and 2019 (64). Though the traditional free-range system is only practiced in poor areas of South Africa, the prevalence of TSTC is reported as the highest from SSA (35). Zambia is identified with the highest point prevalence of porcine cysticercosis (57%), and the second one in terms of pooled prevalence (22%), which is comparable with a report by Shonyela et al. (24.32%) (65). In Zambia, open defecation has been reported as a public health problem (37, 66), particularly in rural areas, where the prevalence of open defecation is reported to be 17% (67).

The subgroup prevalence for Burundi, Uganda, and Mozambique was 15% (within 95% CI range of 6%-26%), followed by Kenya at 13% (95% CI: 7–21) and Tanzania 12% (95% CI: 9–16) (Supplementary Material 4). According to FAOSTAT data (64), Uganda is among the countries that have the largest pig population in Africa, most of which are raised under the traditional husbandry system (42). The country has also the highest per capita consumption of pork in SSA (63). Mozambique is among the countries with the highest prevalence of open defecation at 40%, compared to countries included in this review (67). The pooled prevalence for Kenya and Tanzania were comparable to each other but lower than that reported by Shonyela et al., at 22% (65). The lowest pooled prevalence was recorded in Madagascar (5%) and Rwanda (7%). Madagascar is one of the WHO selected countries for piloting *T*. *solium* control (68).

A subgroup analysis by region revealed a relatively higher pooled prevalence in the Southern region at 22% (95% CI: 15–30), compared to 13% (95% CI: 11–15) in the Eastern region. The high variation on the pooled and sub-group prevalence estimate might be the result of a combination of more than one element capable of affecting the presence of porcine cysticercosis, such as individual host characteristics (e.g., differences in gender, age, and breed), the existence of variation in the exposure to risk factors among and within countries (pig production system, geographical situation, etc.), the environmental conditions or socio-cultural practices enhancing or disfavoring egg dispersal, and survival (16, 69).

The other source of variation might be the inclusion of studies that employed diagnostic techniques with a huge disparity in sensitivity and specificity. A meta-analysis sub-grouped by the diagnostic test estimated the prevalence of 27% (95% CI: 9–50) by carcass dissection, 23% (95% CI: 14–33) by Antibody-based immunodiagnostic techniques, 23% (95% CI: 18–29) by Ag-ELISA, 12% (95% CI: 7–18) by meat inspection, and 9% (95% CI: 7–11) by lingual examination.

Full carcass dissection is a gold standard technique to diagnose porcine cysticercosis (29, 70–72). However, it is not feasible in daily practice because it is extremely laborious, requires trained staff to conduct the procedure, and is expensive as the entire carcass must be sliced in more or less 0.5 cm cuts (72). The prevalence upon carcass dissection in this review is higher than studies in Cameroon (19.6%) and Peru (16.8%) (73).

The lingual examination and meat inspection are reported to be highly specific but poorly sensitive at 7–21% (29, 60, 74) and 22–66% (29, 46, 60), respectively, and likely to lead to underestimation of true prevalence, particularly in light infection. Despite the lower sensitivity of lingual examination, it is widely used for the rapid assessment of porcine cysticercosis in poor endemic areas (17, 23). Meat inspection is used all over the world to ensure the fitness of meat for human consumption, but official guidelines and practices to diagnose porcine cysticercosis vary widely across the countries (70). Some reports highlighted that the prevalence estimation based on meat inspection might be biased because of the pre-screening tradition by traders/butchers during purchasing of pigs; hence, positive pigs are likely not presented for formal slaughter (17, 50, 59).

Most studies included in the review used Ag-ELISA (B158/B60 Ag-ELISA and HP10 Ag-ELISA) to ascertain cases of porcine cysticercosis. Despite the ability of Ag-ELISA for detecting the presence of viable cyst (50), the technique is genus-specific and likely results in overestimation in areas where *Taenia* species (*T. solium, T. hydatigena, T. asiatica*) co-exist (29, 72, 75). Hence, all reports of the prevalence of porcine cysticercosis using Ag-ELISA should be interpreted with care and the results may be more indicative of exposure to *Taenia* spp. broadly. Besides, the result obtained using B158/B60 Ag-ELISA and HP10 Ag-ELISA have not been compared, which could lead to different results given their difference in their diagnostic performance (16).

The sensitivity of HP10 and B158/B60 Ag-ELISAs has been reported to be 44.4–89.5% and 63.3–95.7% (46, 74, 76), while the specificity of the assays has been determined to be 45-100% and 84.4-95% (24, 46, 74, 76), respectively. The prevalence based on Ag-ELISA in the subgroup analysis (23%, 95% CI:18-29) is lower than the report from Burkina Faso, 32.5–48.2% (77), and the Democratic Republic of Congo, 38.4–41.2% (78). The sub-group prevalence of Antibody-based immunodiagnostic techniques in this review (23%, 95% CI: 14–33) was also found lower than 46% in Nigeria (79). The Ab-based immunodiagnostic techniques detect circulating antibodies (Ab) and indicate exposure to the parasite, but not necessarily an active infection (71). However, at the population level, they give a useful indication of areas, where the life cycle of the parasite is ongoing (15, 71). Despite the limitations of the diagnostic tests, we consider the results to indicate the presence and trends of porcine cysticercosis in ESA, and that this information will be useful for targeted research and controlled efforts in the future.

The overall pooled and subgroup prevalence analysis in this review showed high heterogeneity between studies (I^2^> 97%). The presence of publication bias (*p*< *0*.05), which was detected using the funnel plot analysis might be associated with the restriction of the language use, the use of the limited searching platform, the large gap in time frames of data collection (24 years), the would-be biases by the publishers (the lower interest in the publication of manuscripts with statistically non-significant or unfavorable findings), and the authors (research is likely conducted where *T. solium* is a problem and not in other regions).

Thirteen articles out of the 44 selected cross-sectional studies reported statistically significant risk factors for the occurrence of porcine cysticercosis, including lack of latrine at the household level, keeping free-range pigs, semi-confined pig management, home-slaughter, unprotected water source, and old age. The pooled OR of 2.4 was recorded for keeping free-range pigs, and the pooled OR of two was recorded for lack of latrine at household level with insignificant heterogeneity among reports. In SSA, an increased risk of cysticercosis in pigs has been reported to be significantly associated with allowing pigs to roam freely (8, 12, 14, 33, 48), outdoor defecation or lack of latrines at household (30, 48, 56, 80), poor sanitary conditions (15, 81, 82), and age of pigs (4, 15).

Pig rearing is an important livelihood activity in SSA (11), and 60–90% of total pigs in the region are raised under traditional semi-intensive and free-range systems (15). Similarly, about 80% of pigs kept in ESA are raised under the traditional free-ranging system (83). Unhygienic sanitary conditions including limited use or the absence of latrines are prevalent in most rural areas of Africa. According to WHO and UNICEF reports, the overall prevalence of open defecation in SSA was 25% (67), and only 25.7% (23.1–28.6%) of the population in the region has access to improved sanitation (84). In these conditions, tapeworm carriers can disseminate the parasite eggs in their environment and is likely to influence the prevalence of porcine or human cysticercosis (4, 15).

## Conclusion

The data presented in this review described the epidemiology of porcine cysticercosis in the ESA region. The review demonstrated the variability in the reports of porcine cysticercosis. The overall pooled prevalence estimate of porcine cysticercosis in the ESA region based on the included studies was 17%. The evidence concerning porcine cysticercosis in the ESA region provided by the few prevalence studies conducted, so far, showed the magnitude of porcine cysticercosis in pig raising and consumption in parts of the ESA region, providing the impetus for further research, as well as calling for urgent control measures to be implemented in countries where there is enough evidence concerning the presence of porcine cysticercosis. The risk factors which could probably have influenced the transmission and distribution of porcine cysticercosis in the area were: the presence of latrine at the household level, pig management system, water source, and older pig age. The findings will guide in defining priority areas for intervention and control of *T. solium* in the ESA region, but accurate prevalence estimates using more sensitive and specific tests, detailed risk factor analysis incorporating climatic and environmental factors, as well as data on the epidemiology of human cysticercosis and taeniasis, are needed to develop effective control strategies. Epidemiological studies should be promoted in the form of health partnerships and programs implemented within the context of the CWGESA to ensure that comparative results are obtained across the region.

## Data Availability Statement

The original contributions presented in the study are included in the article/Supplementary Material, further inquiries can be directed to the corresponding author/s.

## Author Contributions

YG conceived the idea, developed the review protocol, involved in the critical appraisal of included studies, extracted and analyzed the data, and produced the first draft of the manuscript. HA was involved in the critical appraisal of included studies, extracted, and analyzed the data. TE, NK, EC, and EF were involved in the writing of the manuscript. All authors edited manuscript drafts, provided feedback, and read and approved the final manuscript.

## Funding

This work was part-funded by the Global Challenges Research Fund (GCRF) One Health Regional Network for the Horn of Africa (HORN) Project, from UK Research and Innovation (UKRI) and Biotechnology and Biological Sciences Research Council (BBSRC) (project number BB/P027954/1). Support was also received from the CGIAR Research Program on Agriculture for Nutrition and Health (A4NH), led by the International Food Policy Research Institute (IFPRI). We acknowledge the CGIAR Fund Donors (https://www.cgiar.org/funders/) and the Organization for Women in Science for the Developing World (fund reservation number: 3240303489). The funders had no role in the decision to publish or the preparation of this manuscript. Open access publication fees are supported by the University of Liverpool institutional access fund.

## Conflict of Interest

The authors declare that the research was conducted in the absence of any commercial or financial relationships that could be construed as a potential conflict of interest.

## Publisher's Note

All claims expressed in this article are solely those of the authors and do not necessarily represent those of their affiliated organizations, or those of the publisher, the editors and the reviewers. Any product that may be evaluated in this article, or claim that may be made by its manufacturer, is not guaranteed or endorsed by the publisher.

## Abbreviations

ELISA: Enzyme Linked Immuno Sorbent Assay
ESA: Eastern and Southern Africa
EITB: Enzyme linked Immunoelectro Transfer Blot
FAO: Food and Agricultural Organization
NCC: Neurocysticercosis
SSA: Sub-Sahara Africa
CWGESA: Cysticercosis Working Group for Eastern and Southern Africa
OIE: Office International de-Epizooties
TSTC: Taenia solium taeniosis and cysticercosis
WHO: World Health Organization.
