# Supplementary material for: Epidemiology of Porcine Cysticercosis in Eastern and Southern Africa: Systematic Review and Meta-Analysis
Source: Front Public Health. 2022 Mar 16;10:836177. doi: 10.3389/fpubh.2022.836177 (PMC8966092; doi:10.3389/fpubh.2022.836177)
Supplement: Supplementary file 2 [file Table_2.pdf]

Supplementary Material 5. Critical appraisal assessment result for the selected studies concerning porcine cysticercosis in East and Southern Africa region

Sno	Author	SelectionSelection(5*)				Comparability (2*)		Exposure(3*)		Over all result
		Representativeness of the sample(*)	Non-respondents (*)	Sample size(*)	Ascertainment of the exposure (**)	The study controls for the most important factor (*)	The study control for any additional factor(*)	Assessment of the outcome (2*)	Statistical test(*)	
1	Akoko*et al.*, 2019	*	*	*	*	*		**	*	8
2	Boa *et al.*, 2006	*		*	**	*		*	*	7
3	Braae*et al.*, 2014	*		*	*	*	*	*	*	7
4	Chembensofu*et al.*	*		*	**	*		*	*	7
5	Chilundo*et al.*, 2017	*	*	*	*	*	*	*	*	8
6	Dorny*et al.*, 2004	*	*	*	**	*		**	*	9
7	Eshitera*et al.*, 2012	*		*	*	*	*	*	*	7
8	Fèvre *et al.*, 2017	*		*	*	*		**	*	7
9	Kabululu*et al.*, 2020a	*		*	**	*		**	*	8
10	Kabululu*et al.*, 2015	*		*	*	*	*	*	*	7
11	Kabululu*et al.*, 2020b	*		*	**	*	*	**	*	9
12	Kagira*et al.*, 2010	*		*	*	*	*	*	*	7
13	Komba*et al.*, 2013	*		*	*	*	*	*	*	7
14	Krecek*et al.*, 2008	*		*	*	*	*	**	*	8
15	Krecek *et al.*, 2012	*	*	*	*	*	*	**	*	9
16	Kungu*et al.*, 2017	*		*	*	*	*	*	*	8
17	Thomas, 2013	*			*	*	*	*	*	7
18	Kisakye and Masaba, 2002	*	*		*			*	*	5
19	Maganira*et al.*, 2019	*		*	*	*	*	**	*	8
20	Matos *et al.*, 2011	*	*	*	*		*	*	*	7
21	Minani*et al.*, 2021	*	*	*	*	*	*	**	*	9
22	Mkupasi*et al.*, 2011	*	*	*	*		*	*	*	7
23	Mushonga*et al.*, 2018	*		*	*	*	*	*	*	7

24	Mutua *et al.,* 2007	*	*	*	*		*	*	*	7
25	Newell *et al.*, 1997	*			*	*	*	**	*	7
26	Ngowi*et al.*2010	*	*	*	*		*	*	*	7
27	Ngowi*et al.*, 2004a	*	*	*	*	*	*	**	*	9
28	Ngowi*et al.,* 2004b	*	*		*	*	*	*	*	7
29	Nguhiu*et al.,* 2017	*	*	*	*	*		*	*	7
30	Nsadha*et al.*, 2014	*	*	*	*			**	*	7
31	Phiri *et al.,* 2002	*	*	*	*			**	*	7
32	Phiri *et al.,* 2006	*	*		**			**	*	7
33	Pondja*et al.,* 2010	*		*	*	*	*	**	*	8
34	Pondja*et al.,* 2015	*	*	*	*	*	*	*	*	8
35	Shongwe*et al.,* 2020	*		*	*	*	*	*	*	7
36	Porphyre et al., 2015	*	*	*	*		*	*	*	7
37	Porphyre*et al.,* 2015	*		*	*	*	*	*	*	7
38	Shonyela*et al.,* 2017	*		*	*	*	*	*	*	7
39	Sikasunge*et al.,* 2007	*		*	*	*	*	*	*	7
40	Sikasunge*et al.,* 2008	*		*	*	*	*	*	*	8
41	Thomas *et al.,* 2016	*	*	*	**	*		*	*	8
42	Waiswa*et al.,* 2009	*	*	*	*	*		*	*	7
43	Wardrop*et al.*, 2015	*	*		*	*	*	**	*	8
44	Yohana *et al.,* 2013	*	*	*	*	*		*	*	7
45	Zirintunda and Ekou, 2015	*	*	*	*	*		*	*	7

Newcastle - Ottawa Quality Assessment Scale (NOS) for cohort studies and NOS adapted for cross-sectional studies were employed to assess the quality of included studies. NOS score of 7 or more considered a “good” study.
NB. Kisakye and Masaba, 2002 excluded from the review because it score below 7
