# Supplementary figures and images for: Epidemiology of Porcine Cysticercosis in Eastern and Southern Africa: Systematic Review and Meta-Analysis
Source: Front Public Health. 2022 Mar 16;10:836177. doi: 10.3389/fpubh.2022.836177 (PMC8966092; doi:10.3389/fpubh.2022.836177)

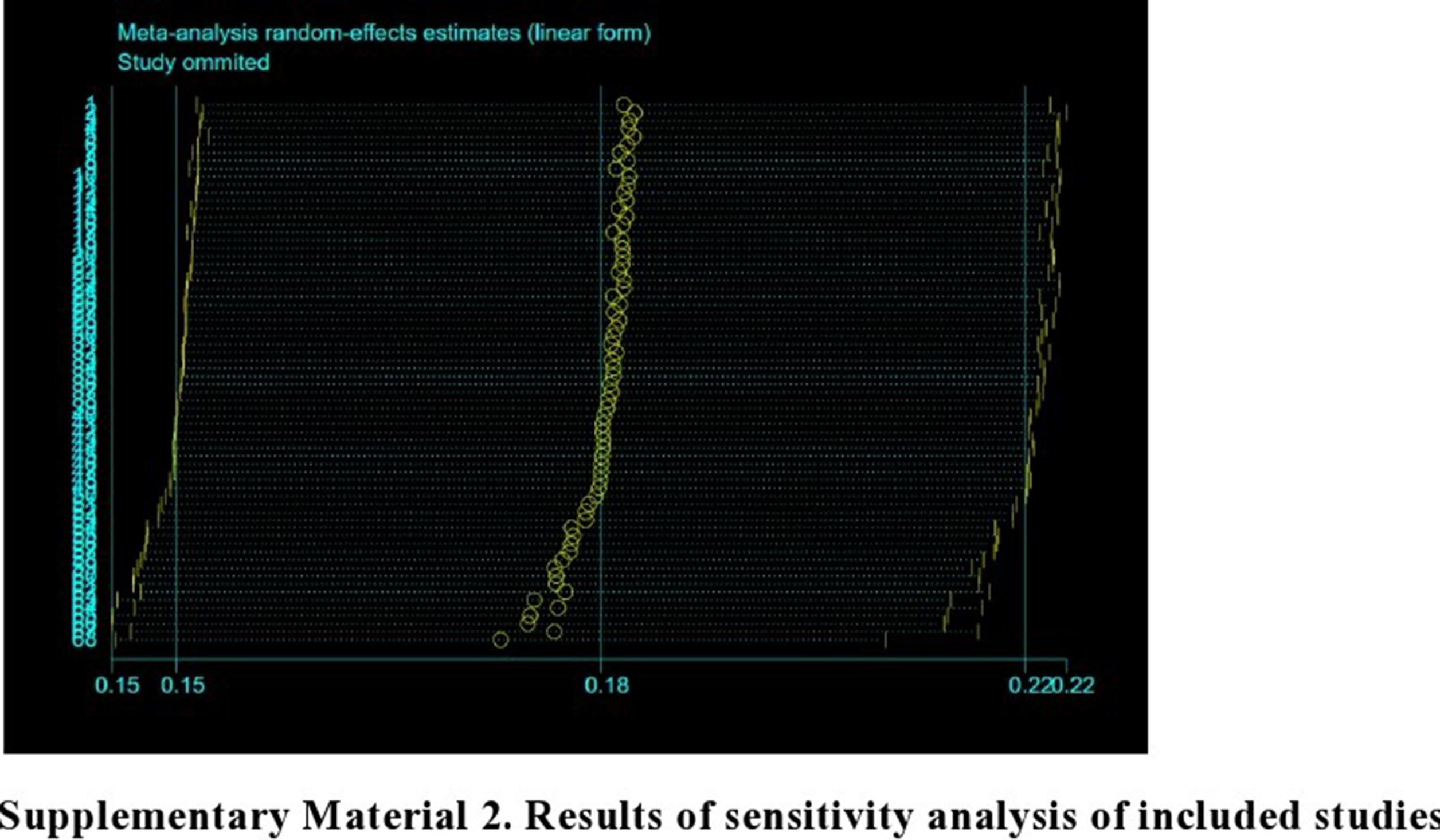

Supplement: Supplementary file 3 [file Image_1.jpg]

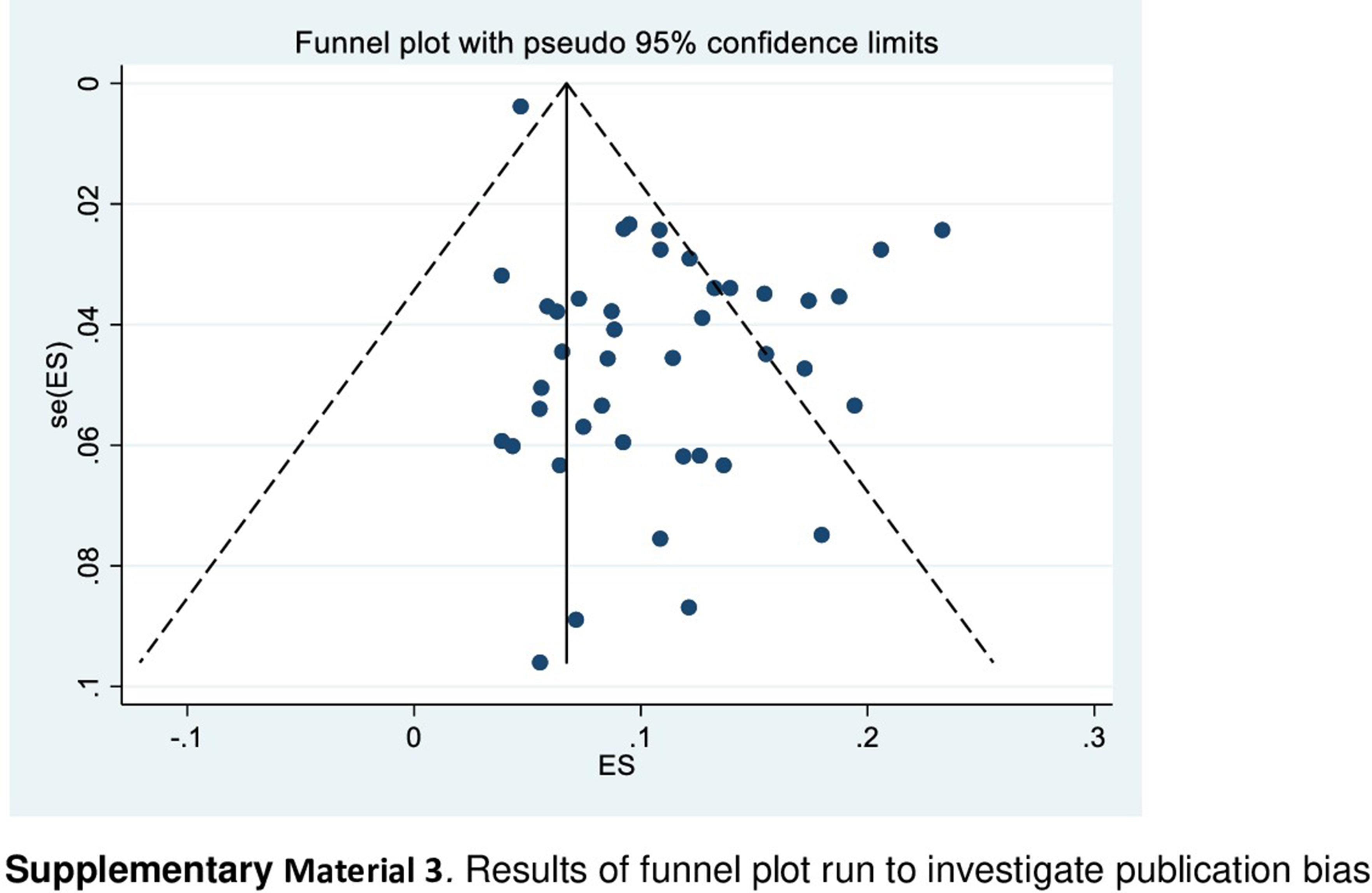

Supplement: Supplementary file 4 [file Image_2.jpg]

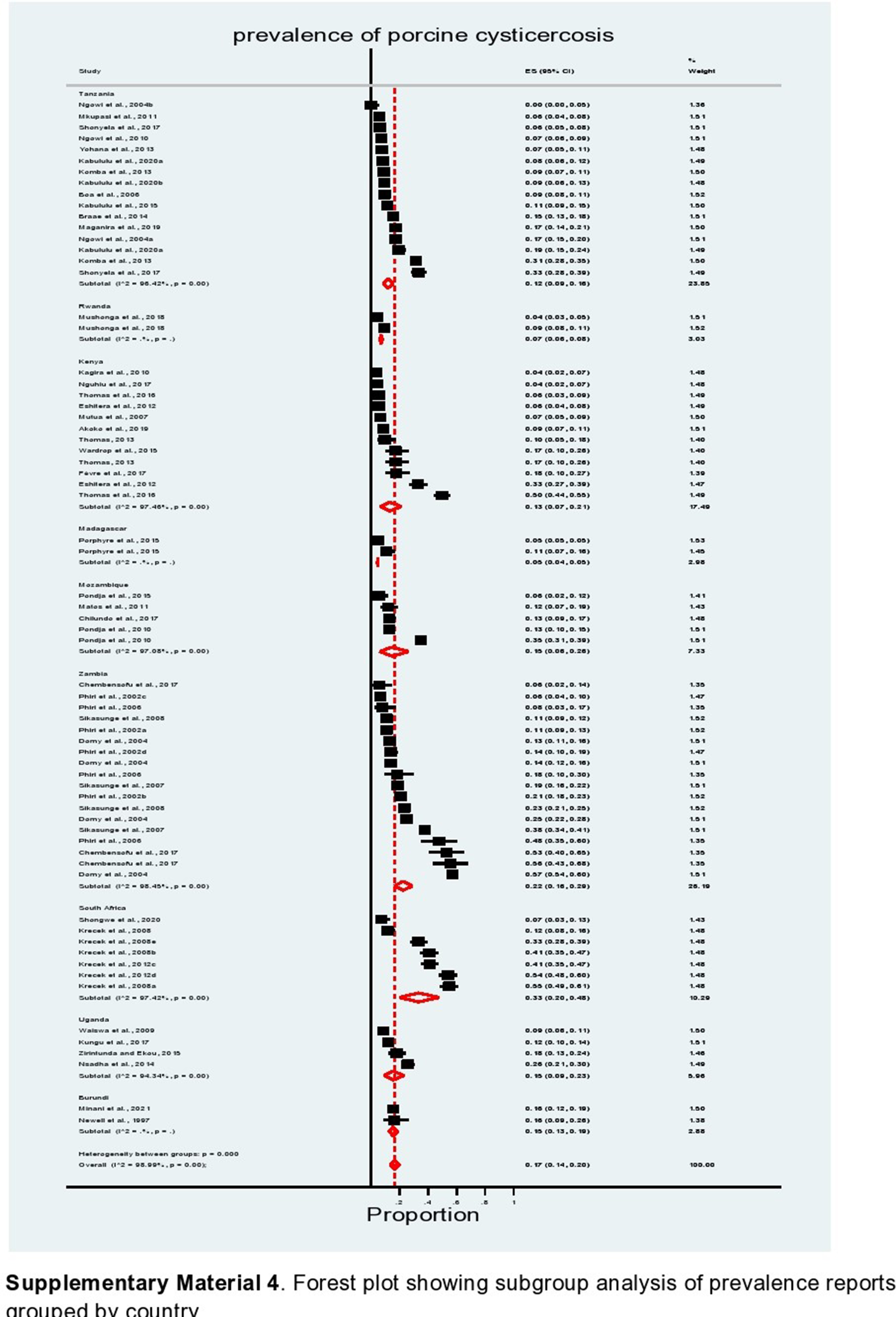

Supplement: Supplementary file 5 [file Image_3.jpg]
